# Nonlinear Saturable and Polarization-induced Absorption of Rhenium Disulfide

**DOI:** 10.1038/srep40080

**Published:** 2017-01-05

**Authors:** Yudong Cui, Feifei Lu, Xueming Liu

**Affiliations:** 1 State Key Laboratory of Modern Optical Instrumentation, Department of Optical Engineering, Zhejiang University, Hangzhou 310027, China; 2 State Key Laboratory of Transient Optics and Photonics, Xi’an Institute of Optics and Precision Mechanics, Chinese Academy of Sciences, Xi’an 710119, China; 3 School of Physics and Electronic Science, Hunan University of Science and Technology, Xiangtan 411201, PR China

## Abstract

Monolayer of transition metal dichalcogenides (TMDs), with lamellar structure as that of graphene, has attracted significant attentions in optoelectronics and photonics. Here, we focus on the optical absorption response of a new member TMDs, rhenium disulphide (ReS_2_) whose monolayer and bulk forms have the nearly identical band structures. The nonlinear saturable and polarization-induced absorption of ReS_2_ are investigated at near-infrared communication band beyond its bandgap. It is found that the ReS_2_-covered D-shaped fiber (RDF) displays the remarkable polarization-induced absorption, which indicates the different responses for transverse electric (TE) and transverse magnetic (TM) polarizations relative to ReS_2_ plane. Nonlinear saturable absorption of RDF exhibits the similar saturable fluence of several tens of μJ/cm^2^ and modulation depth of about 1% for ultrafast pulses with two orthogonal polarizations. RDF is utilized as a saturable absorber to achieve self-started mode-locking operation in an Er-doped fiber laser. The results broaden the operation wavelength of ReS_2_ from visible light to around 1550 nm, and numerous applications may benefit from the anisotropic and nonlinear absorption characteristics of ReS_2_, such as in-line optical polarizers, high-power pulsed lasers, and optical communication system.

The booming exploration of nanomaterials, especially the two-dimensional (2D) materials, helps the promotion of the investigation and application in optoelectronics and photonics^1^,^2^,^3^,^4^. Because of the broadband absorption, ultrafast carrier dynamics and large third-order nonlinear susceptibility, various nanomaterials have been demonstrated to be the ideal nonlinear optical materials^5^,^6^,^7^,^8^,^9^,^10^,^11^. Graphene has a single atomic layer of hexagonal lattice formed with sp^2^-hybridized carbon that can be described in terms of massless Dirac fermions^12^. The gapless linear dispersion of the Dirac electron maintains the wavelength-independent absorption for graphene whose nonlinear optical response has been also demonstrated in a broad operation bandwidth^5^,^13^,^14^,^15^,^16^. Motivated by the significant development of graphene, the saturable absorption of transition metal dichalcogenides (TMDs) has also attracted increasing attentions because of their enhanced broadband and ultrafast nonlinear optical responses, such as molybdenum disulfide (MoS_2_), tungsten disulphide (WS_2_) and molybdenum diselenide (MoSe_2_)^6^,^17^,^18^,^19^,^20^.

Layered TMDs with the chemical formula MX_2_ is generally composed of a plane of hexagonally arranged transition metal atoms (M) sandwiched between two hexagonal planes of chalcogen atoms (X)^21^,^22^. The intralayer M and X are bonded covalently, whereas the interlayers are coupled via weak van der Waals forces^22^. Due to the interlayer coupling, the bandgap is transformed from indirect to direct as the bulk TMDs are decreased to monolayer^21^,^22^,^23^. These features render them as the promising candidate for optoelectronic and photovoltaic devices^22^,^24^,^25^. The present most studied monolayer TMDs have a 2 H lattice structure, which means that their electronic and optical properties are in-plane isotropic^21^,^26^. Recently, in-plane anisotropic 2D materials, such as black phosphorus (BP), have been demonstrated for the applications in optoelectronics and photonics^27^,^28^,^29^. BP shows anisotropic electronic and optical properties along the armchair and zigzag direction as a result of the orthorhombic crystal structure^28^,^30^. The bandgap of BP can be widely tuned with the variation of layers, which could be employed in broadband application^30^,^31^,^32^. However, BP displays weak environmental stability. The degradation and breakdown would occur due to the photo-oxidation and the absorption of water during several hours to days^27^,^33^.

Here, we study a new member of the TMDs family, rhenium disulphide (ReS_2_), whose layered material also possesses strong in-plane anisotropic, but exhibits much better environmental stability than BP^29^,^34^. ReS_2_ has been demonstrated to have a unique distorted 1 T structure with weak interlayer coupling^29^. As a result, both the monolayer and bulk ReS_2_ are direct-bandgap semiconductors and have nearly identical band structures with the bandgap of ~1.35 eV (bulk) and ~1.43 eV (monolayer)^34^,^35^. This is exactly different from the conventional TMDs whose band structures are strongly dependent on the number of layer^21^,^22^,^34^,^35^. The previous researches on ReS_2_ mainly concentrated on its electronic properties and linear optical responses by implementing the transistors and photodetectors^34^,^36^. ReS_2_-based photodetectors exhibit the high external quantum efficiency and photoresponsivity with the probe wavelength of 633 nm^36^. Recently, the anisotropic transient absorption was measured for ReS_2_ around 800 nm corresponding to its bandgap^37^. However, till now, ReS_2_ was mainly studied and applied at wavelengths less than 830 nm, and its nonlinear optical response remains unclear. The nonlinear saturable and polarization-induced absorptions of ReS_2_ are demonstrated at infrared communication wavelength in our work. ReS_2_ is transferred onto a side-polished surface of optical fiber, where light propagates parallel to the ReS_2_ layer in one direction. The ReS_2_-covered D-shaped fiber (RDF) displays the remarkable polarization-induced absorption, while the nonlinear saturable absorption possesses the features that are independent of polarization. The performances of RDF are further verified in an erbium-doped fiber (EDF) laser in which ultrafast pulses are generated at 1564 nm. This work demonstrates the optical performance of ReS_2_ at near-infrared region beyond its bandgap.

## Experimental Results

### Characterization of ReS_2_-covered D-shaped fiber

**Figure 1:**
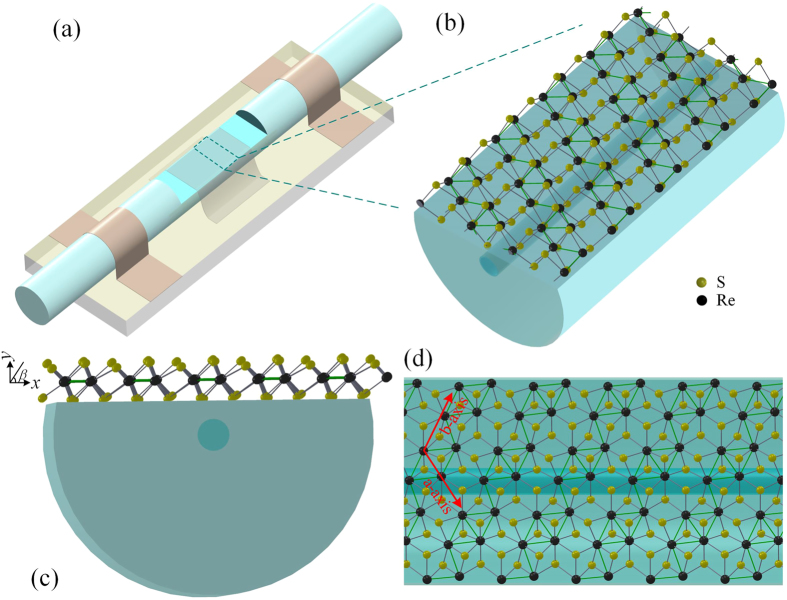
Schematic diagrams of (**a**) ReS_2_-covered D-shaped fiber (RDF) saturable absorber (SA), (**b**) zoom-in image of D-shaped area, (**c**) cross-section, and (**d**) top view of RDF SA.

D-shaped fiber is produced by polishing a side of optical fiber into the fiber core, as shown in Fig. 1(a). ReS_2_ is produced via chemical vapor deposition (CVD) grown on sapphire substrate, which is cut into strip samples with the width of ~1 mm and the length of ~5 mm. The ReS_2_ sample is transferred from the substrate onto the D-shaped fiber via the wet transfer method like that of graphene^2^,^38^. Firstly, ReS_2_ coated with polymethylmethacrylate (PMMA) is cut into strip samples. Then ReS_2_ strips are separated from the substrate in the etchant that is removed by rinsing several times in the deionized (DI) water. Finally, ReS_2_ strips are transferred onto the side-polished area in DI water. The detailed fabrication procedure of the RDF SA is provided in the experimental section. The schematic diagram of RDF SA is shown in Fig. 1(a). Figure 1(b) is the schematic of the D-shaped fibre covered with ReS_2_, which can be regarded as the zoom-in image of Fig. 1(a). Figure 1(c,d) are the side and top view of Fig. 1(b), respectively. It can be seen the crystal structure of monolayer ReS_2_, which illustrates a distorted 1 T structure. Re atoms form zigzag chains along the b-axis shown in Fig. 1(d), which is expected to induce the in-plane anisotropy^29^,^34^. In Figure 1(c), the polarization angle *β* in x-y plane is defined as the angle between the polarization direction of light and the ReS_2_ layer. And x-y plane is perpendicular to the light transmission direction. For *β* = 0°, the light polarization direction is parallel to ReS_2_ plane. In this case, it is defined as transverse electric (TE) wave. For *β* = 90°, the light polarization is perpendicular to ReS_2_ plane, which is defined as transverse magnetic (TM) wave.

**Figure 2:**
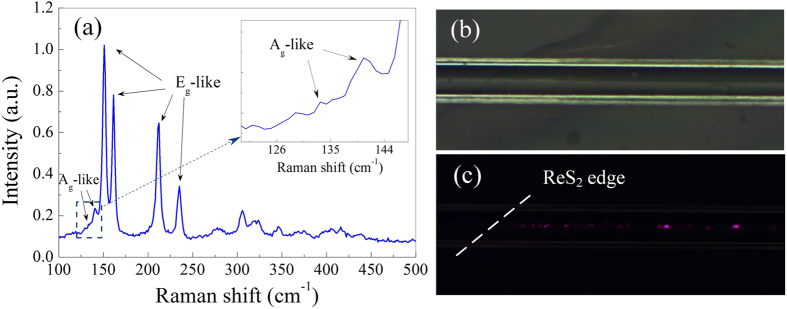
(**a**) Raman spectrum of ReS_2_. Microscopy photograph of the D-shaped fiber covered with ReS_2_ (**b**) before and (**c**) after injecting 632.8 nm laser. White dash line indicates the edge of ReS_2_ film.

The samples are characterized by Raman spectrometry with the excitation laser wavelength of 633 nm. The non-resonance Raman scattering measurement result is shown in Fig. 2(a). Because of the low crystal symmetry and the coupling among fundamental Raman modes, more Raman shift peaks can be observed^34^. The four prominent Raman peaks around 150, 161, 212 and 235 cm^−1^ correspond to the in-plane vibration modes (Eg-like) of Re atom and the low frequency peaks around 134 and 140 cm^−1^ correspond to the out-of-plane vibration modes (Ag-like)^29^,^39^. However, its Raman spectrum is insensitive to the layer number since ReS_2_ is electronically and vibrationally decoupled^29^. And as ReS_2_ is a kind of in-plane anisotropic 2D material, the line widths and the peak intensities of all the Raman peaks vary with the polarization of probe laser^39^. The layer number can only be identified with the slight frequency difference of specific Raman modes^40^. The spacing between the first peaks of Eg-like (133.4 cm^−1^) and Ag-like (150.7 cm^−1^) is ~17.3 cm^−1^, which indicates the monolayer structure^40^. It should be noted that the used ReS_2_ is not uniform and is composed of monolayer and multilayer structures. However, the similar Raman results like that in Fig. 2(a) can be obtained for the most areas on the sample. So it could be inferred that the majority of the used ReS_2_ film is monolayer. Figure 2(b,c) show the optical microscopy images of the transferred PMMA/ReS_2_ film on the polished surface of the D-shaped optical fiber. The areas on the both sides of D-shaped fiber are the transition of the film from polished surface to substrate. The edge of film can be observed in Fig. 2(b), which is marked in the corresponding microscopy image (Fig. 2(c)) with reduced lighting after injecting 632.8 nm laser. It can be clearly differentiated with the dividing line that no red radiation can be observed without ReS_2_ and a small amount of evanescent field leak out with ReS_2_ film.

### Polarization-induced absorption

**Figure 3:**
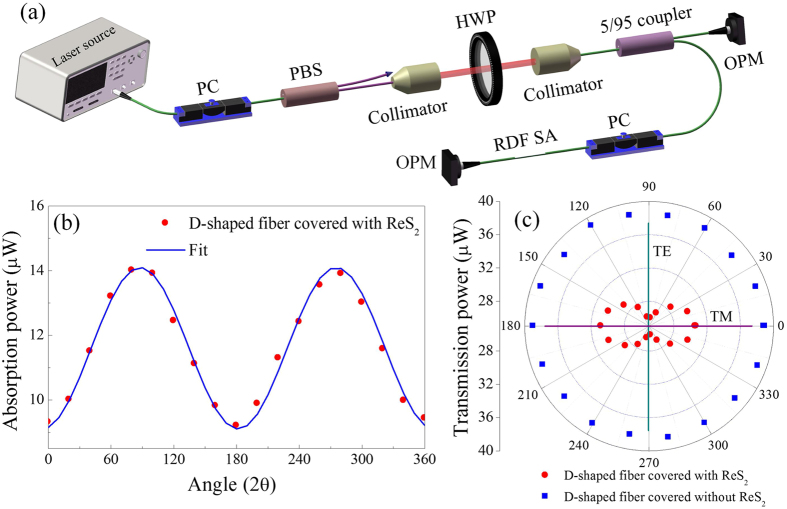
Polarization-induced absorption measurement. (**a**) Experimental setup for measuring the polarization-induced loss. PC: polarization controller; PBS: polarization beam splitter; HWP: half-wave plate; OPM: optical power meter. (**b**) Absorption power of RDF SA as a function of the polarization angle of the incident laser. The experimental data is fitted with solid curve. The equation that produces the best fit is as P_a_ = −2.5∙cos(0.96∙2θ + 0.2)+11.6. P_a_ is the absorption power. θ is the rotation angle of HWP and 2θ is the corresponding rotation angle of linear polarized laser. (**c**) The corresponding transmission power of D-shaped fiber covered with (dots) and without (squares) ReS_2_ in polar coordinate.

The polarization-induced absorption of the D-shaped fiber covered with and without ReS_2_ is performed with the experimental setup shown in Fig. 3(a). The linear polarization laser centered at 1550 nm outputted from a polarization beam splitter (PBS) is rotated from 0° to 360° by a half-wave plate. The detailed description about the experimental setup is provided in the experimental section. As shown in Fig. 3(b), the absorption power of RDF SA varies periodically with the polarization angle, which is fitted well with a cosine function. Figure 3(c) shows the corresponding transmission power of D-shaped fiber covered with and without ReS_2_ in a polar coordinate. It can be obviously observed the ReS_2_-light interaction behavior with the polarization angles. For the TE and TM polarizations relative to the ReS_2_ plane, it shows the maximum and minimum absorption, respectively. The polarization-dependent performance should be attributed to the presence of ReS_2_. However, no obvious polarization-induced loss can be observed for D-shaped fiber without ReS_2_ in our experiments and in previous reports before the graphene transference^38^,^41^. Without ReS_2_, the loss of the D-shaped optical fiber is ~0.5 dB, while the losses for TE and TM polarizations are 4.4 and 2.6 dB with ReS_2_, respectively.

### Nonlinear saturable absorption

**Figure 4:**
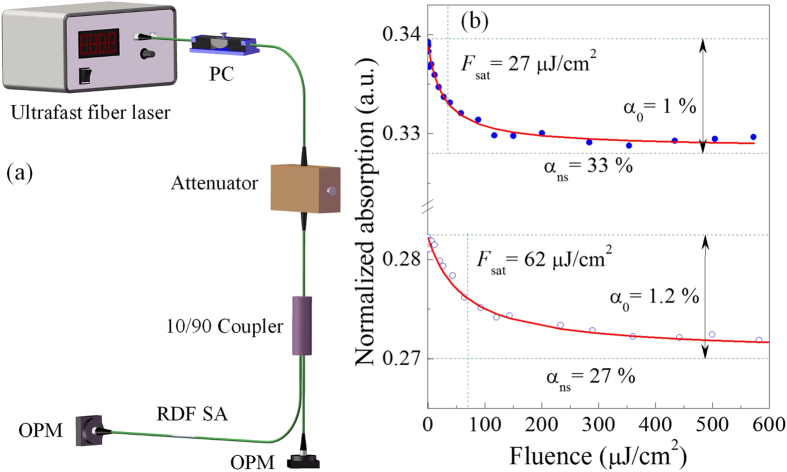
Nonlinear optical characterization of RDF SA. (**a**) Schematic diagram of experimental setup. (**b**) Nonlinear saturable absorptions measured with ultrafast pulses at two orthogonal polarizations corresponding to TM (below) and TE (up) polarizations, respectively. The solid curves are the fit to the experimental data.

The nonlinear saturable absorption of RDF SA is measured with ultrafast pulses at different polarization angles. The experiment setup is illustrated in Fig. 4(a) where a homemade ultrafast fibre laser centered at 1560 nm with the repetition rate of ~8 MHz is used as the laser source and an attenuator is used to control the input power. Two optical power meters (OPMs) are utilized to monitor the optical power from two branches divided by a 90/10 coupler. A PC is placed before the attenuator to adjust the polarization of input ultrafast pulses, and the loss of RDF SA changes with the input pulse polarization. Figure 4(b) shows the typical nonlinear saturable absorptions as a function of optical fluence with the maximum and minimum nonsaturable absorption which reflect the nonlinear responses of TE and TM polarized pulses, respectively. The experimental data are fitted on the basis of a simplified two-level saturable absorption model^5^,^42^:


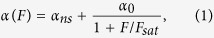


where *α(F*) is the intensity-dependent absorption coefficient, and *α*_0_, *α*_ns_ and *F*_*sat*_ are the linear limit of saturable absorption, nonsaturable absorption, and saturation fluence, respectively. When Eq. (1) fits well with the experimental data, the corresponding *α*_0_, *α*_ns_ and *F*_*sat*_ can be obtained. With the lower nonsaturable absorption, the linear limits of the saturable absorption (α_0_) and the saturation fluence (*F*_sat_) are approximately 1.2% and 62 μJ/cm^2^, respectively. With the higher nonsaturable absorption, it shows a smaller saturation fluence (~27 μJ/cm^2^), while the modulation depth (~1%) is equivalent to that with the lower nonsaturable absorption.

### Application of RDF SA on ultrafast fiber lasers

**Figure 5:**
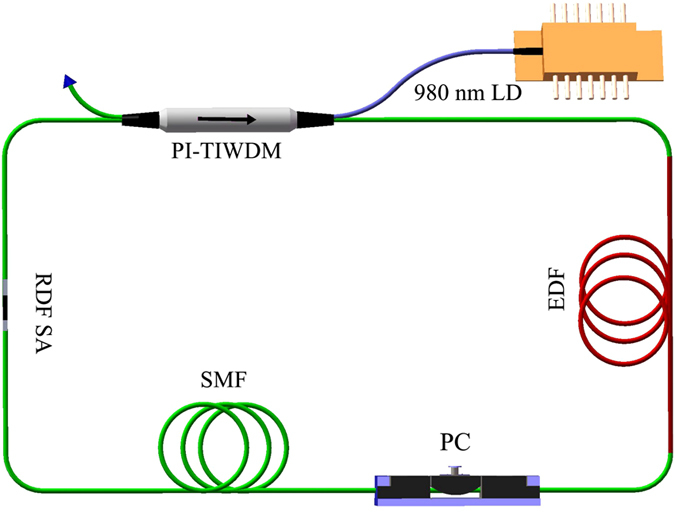
Laser setup. EDF, erbium-doped fiber; PI-TIWDM, polarization-independent tap-isolator-wavelength-division multiplexer; PC, polarization controller; LD, laser diode; SMF, single-mode fiber; RDF SA, ReS_2_-covered D-shaped fiber saturable absorber.

A schematic of the fiber laser mode-locked by ReS_2_ is shown in Fig. (5). The fiber laser system is composed of a 5-m-long erbium-doped fiber (EDF), a RDF SA, a polarization controller (PC), a section of standard single-mode fiber (SMF), and polarization-independent tap-isolator-wavelength-division multiplexer (PI-TIWDM). The PI-TIWDM combines the functions of wavelength-division multiplexer, optical coupler and isolator.

**Figure 6:**
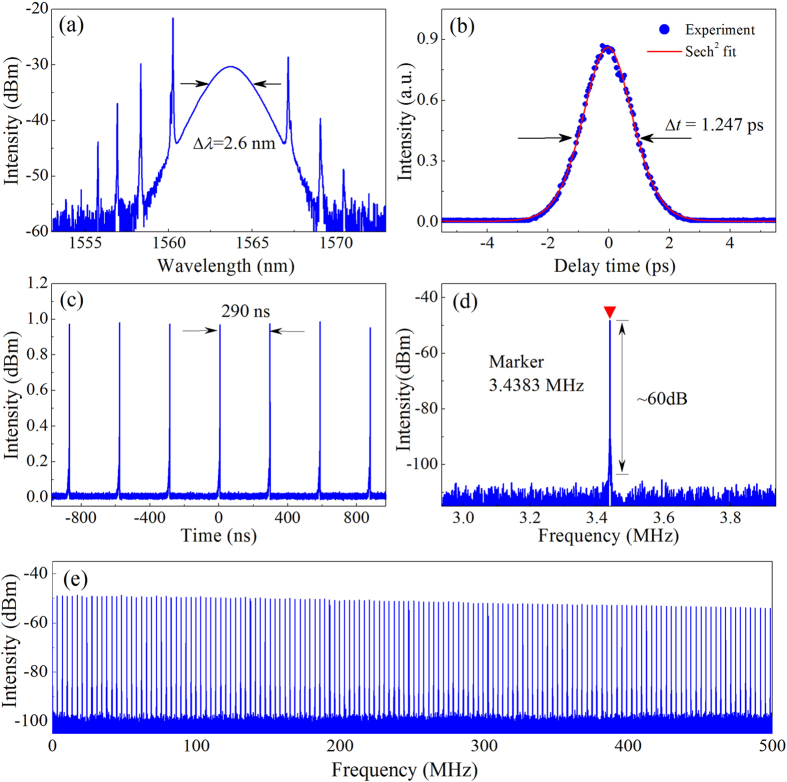
Typical laser characteristics. (**a**) Optical spectrum with a spectral resolution of 0.02 nm. The spectral width Δ*λ* is approximately 2.6 nm. (**b**) Autocorrelation trace of the experimental data (dots) and Sech^2^-shaped fit (solid curve). (**c**) Oscilloscope trace with a pulse separation of ~290 ns, corresponding to the cavity length of ~59 m. (**d**) Fundamental radio-frequency (RF) spectrum with a span of 1 MHz. (**e**) Wideband RF spectrum up to 500 MHz.

In the RDF SA-based Er-doped fiber laser, continuous wave (CW) is achieved at a pump power less than 5 mW, which benefits from the low loss of RDF SA. By increasing the pump power to ~20 mW, self-started mode locking operation is achieved. However, the mode-locking operation is sensitive to the state of PC since the RDF SA introduces a polarization-dependent absorption. In fact, mode locking operation can be self-started at ~8 mW with the appropriate state of PC. At this time, there are multiple pulses circulating in the laser cavity. The hysteresis phenomenon exits in the mode-locked fiber laser^43^. The single pulse operation can be achieved when the pump power is gradually decreased from ~20 mW to ~5 mW, but it is hard to observe the single pulse operation by increasing pump power. Figure 6 characterizes the typical output pulses in the experiment. As shown in Fig. 6(a), the central wavelength and spectral bandwidth of the optical spectrum are ~1564 nm and ~2.6 nm, respectively. Several pairs of sidebands are distributed on the both sides of spectrum, which is the typical characteristics of standard soliton pulses^2^,^9^. The laser cavity contains ~5 m EDFs and ~54 m SMFs, and the total cavity dispersion is calculated as −1.1 ps^2^. Under the net negative dispersion, conventional soliton can generate due to the balance between dispersion and nonlinearity of fiber^2^,^15^. The autocorrelation trace of the output pulse is shown in Fig. 6(b). By assuming a sech^2^ profile, a full width at half maximum is ~1.9 ps, and the deconvolution yields the pulse duration of 1.25 ps. TBP is calculated as 0.4, which means that the pulses are slightly chirped. The pulse train shown in Fig. 6(c) illustrates that the separation between adjacent pulses is ~290 ns corresponding with the cavity round-trip time. The radio-frequency (RF) spectrum with a span of 1 MHz is shown in Fig. 6(d). The fundamental repetition rate of DS is ~3.43 MHz with the signal-to-noise ratio of about 60 dB, implying a low-amplitude fluctuation and good mode-locking stability. A wideband RF spectrum up to 500 MHz is shown in Fig. 6(e) where no spectrum modulation can be observed indicating no Q-switching instabilities^2^.

## Discussions

Single layer of TMDs with lamellar structures like that of graphene have received much attention because some of them are semiconductors with sizable bandgaps^21^. For instance, the monolayer MoS_2_ has a direct bandgap of ~1.8 eV and the bandgap of the bulk MoS_2_ is indirect with the range of 0.9–1.3 eV^6^,^18^,^21^. Optical absorption is closely related to the electronic band structure. The electronic band structure of ReS_2_ has been reported in numerous previous studies^29^,^34^. The monolayer, multilayer and bulk ReS_2_ have the nearly identical band structure with the direct bandgap of ~1.4 eV^29^. It seems that TMDs semiconductors are beyond the operation bandwidth as a saturable absorption device in Yb-, Er- and Tm-doped fiber lasers. However, numerous studies have revealed that MoS_2_ can be used as SA in a broad operation wavelength^18^,^19^. Various theories have been proposed to interpret the experimental observations, such as defect-induced sub-band and edge states of the materials^6^,^23^,^44^. Similarly, Horzum *et al*. theoretically investigate the atomic defects in monolayer ReS_2_, and the densities of states of monolayer ReS_2_ with different atomic defects were calculated^45^. It is found that the formation of the S vacancy reduces the bandgap from 1.43 to 1.08 eV and the bandgap with Re defects becomes only 0.35 eV^45^. The saturable absorptions illustrated in our works are around 1550 nm (~0.8 eV) which is beyond the bandgap of ReS_2_ (~1.4 eV). The results should originate from the atomic defect in ReS_2_, because the imperfections formation, e.g., point defects and grain boundaries, are unavoidable during the growth and transfer process.

In previous works, SAs were generally fabricated by placing the nanomaterials between two fiber ends, which limits the interaction distance^15^,^30^,^46^. To enhance the light-graphene interaction, evanescent field interaction schemes based on a microfiber were implemented which can take full advantage of the nonlinear absorption of graphene^2^,^47^,^48^. D-shaped fiber having a more robust structure than microfiber, is an alternative to lengthen the interaction distance and improve the damage threshold^8^,^38^,^49^. For ReS_2_, D-shaped fiber-based evanescent field interaction scheme provides a platform to investigate the in-plane characteristics, as light propagates parallel to the ReS_2_ layer in one direction. Figure 3(b,c) show a polarization-induced absorption that the losses for TE and TM polarization waves are 4.4 and 2.6 dB, respectively. The results indicate that the optical responses of ReS_2_ are different for TE and TM polarizations^38^. The similar performances of graphene have been discussed, which was used to achieved the in-line polarizer and modulator^41^,^49^. The polarization effect in graphene-waveguide structures originates from the different optical absorption in graphene for TE and TM radiations^41^. However, because of the polarization-dependent loss induced by D-shaped fiber scheme, nonlinear polarization rotation (NPR) effect may exit in the laser system. Although the state of PC can influence the characteristics of pulses, NPR hardly dominate the mode-locking dynamics. If the RDF SA component is excluded from the proposed fiber laser, it fails to initiate the mode-locking operation. In fact, for any polarization direction RDF SA exhibits the saturable absorption with the similar modulation depth which can been seen in Fig. 4(b). As a result, self-started mode locking can always be achieved when adjusting PC.

Although graphene and MoS_2_ have been reported to possess the polarization-dependent absorption, ReS_2_ displays some unique properties. As the monolayer and multilayer ReS_2_ have the similar direct bandgap, the experimental results are insensitive to the thickness of sample. In contrast, only monolayer graphene and MoS_2_ have the direct bandgap, and their band structures vary with the thickness^12^,^21^,^22^. As a result, the operation wavelength and absorption intensity would change obviously with the thickness. The nonlinear saturable absorption of ReS_2_ shows the similar merits as discussed above. For example, the saturable intensity of monolayer MoS_2_ is about several tens of MW/cm^2^, while it increases at least ten times for multilayer MoS_2_^6^,^17^. For monolayer and multilayer ReS_2_, the characteristics of saturable absorption would be identical. In addition, the saturable absorptions for two orthogonal polarization pulses were rarely measured in the D-shaped fiber scheme. It is significant for the development of saturable absorber based on D-shaped fiber to show that RDF exhibits the similar saturable fluence and modulation depth for ultrafast pulses with two orthogonal polarizations.

ReS_2_ maintains the characteristics that the bandgap does not change much from monolayer to bulk state. As a result, ReS_2_ with the different thicknesses display the similar optical properties. The influence of the thickness on the results in the experiment is discussed as follows. Firstly, the similar experimental results could be achieved with shorter length of ReS_2_ film when the thicker ReS_2_ sample is used, because it possesses the stronger absorption than monolayer ReS_2_. But the linear loss would also increase due to the larger perturbation to the evanescent field. When the ReS_2_ with too large thickness is used, the results cannot be achieved. Secondly, the fabrication of perfect monolayer ReS_2_ require the strict growth condition, such as time, temperature, catalyst *et al*., while the grown of thin ReS_2_ film without thickness limitation needs much lower condition. Finally, here the performance of ReS_2_ around 1550 nm depends on the atomic defect which is related to the growth condition. However, multilayer ReS_2_ with different growth condition may obtain the distinct results.

In conclusion, we investigated the linear and nonlinear optical responses of ReS_2_ experimentally with assistant of D-shaped fiber. The linear absorption of RDF changes periodically with the polarization angle, which indicates the in-plane different absorptions of ReS_2_ for TE and TM polarizations. The optical responses to different polarized ultrafast pulses exhibit the similar features, such as the modulation depth and the saturable fluence, although it exhibits the polarization-induced nonsaturable absorption. The nonlinear absorption of RDF is applied to the ultrafast fiber laser as a SA. Self-started mode-locking operation can be always achieved, which is independent of the state of PC. The results confirm the excellent performance of RDF as a saturable absorption device, which has significant potential applications in a wide wavelength range from visible to mid-infrared (~0.35 eV).

### Experimental Sections

#### Preparation of the ReS_2_-covered D-shaped fiber SA

ReS_2_ is produced by chemical vapor deposition (CVD) grown on sapphire substrate. The optical fiber is side-polished into the fiber core with the distance from fiber core to the surface of ~2 μm and the polishing length of ~20 mm, which is fixed on a glass substrate with the polished surface upward as shown in Fig. 1(a). Before transfer of ReS_2_, polymethylmethacrylate (PMMA) is spin-coated uniformly onto ReS_2_ and dried for several hours. Then it is cut into strip samples with the width of ~1 mm and the length of ~5 mm. The samples are put into the etchant solution to detach ReS_2_ from the substrate. The resulted PMMA/ReS_2_ strips are transferred into deionized (DI) water three times to rinse the etchant and residues. Subsequently, the side-polished fiber is immerged into the DI water where the ReS_2_ strips are floating. The position and direction of the sample are carefully controlled with a probe. When the strip is placed on the top of the side-polished area, the D-shaped fiber is lifted out of the DI water, and PMMA/ReS_2_ spontaneously covered on it. The schematic of ReS_2_-clad microfiber is shown in Fig. 1. Figure 2(b,c) show the optical microscopy image of the transferred ReS_2_/PMMA film on the polished side of the D-shaped optical fiber.

#### Polarization-induced absorption experiment

The polarization-induced absorption is implemented with the setup shown in Fig. 3(a). The laser source is from a home-made mode-locked fiber laser at 1550 nm. The polarization beam splitter (PBS) is used to divide the output laser into two orthogonal beams whose intensity can be optimized with a polarization controller (PC). After that, the linear polarized laser is collimated to pass through a half-wave plate (HWF) to control the polarization direction, and then is coupled to an optical coupler via another collimator. 5% port is used to detect the input power and 95% port is connected to RDF, which can eliminate the effect of the fluctuation of laser source. The absorption as a function of polarization angle is obtained by comparing the output powers from two ports.

#### Ultrafast fiber laser experiment

A schematic of the fiber laser mode-locked by ReS_2_ is shown in Fig. 5. The fiber laser system is composed of a 5-m-long erbium-doped fiber (EDF) with 3 dB/m absorption at 980 nm, which is pumped by a 980 nm laser diode (LD) via a polarization-independent tap-isolator-wavelength-division multiplexer (PI-TIWDM). PI-TIWDM can also function as an output coupler with a ratio of 10% and a polarization-independent isolator to force unidirectional propagation. The intracavity polarization controller (PC) is used to adjust the cavity linear birefringence to optimize the mode-locking performance. The RDF SA is used to initiate the mode-locking operation. The EDF and SMF have dispersion parameters of approximately −25 and 17 ps/(nm·km) at 1550 nm, respectively. The cavity length is ~59 m including ~5 m EDFs and ~54 m SMFs. The total cavity dispersion is ~−1.1 ps^2^.

#### Measurement method

An optical spectrum analyzer (Yokogawa AQ-6370), an autocorrelator, a 6-GHz oscilloscope, a radio-frequency (RF) analyzer, and a 3-GHz photodetector are used to measure the laser output performance.

## Additional Information

**How to cite this article**: Cui, Y. *et al*. Nonlinear Saturable and Polarization-induced Absorption of Rhenium Disulfide. *Sci. Rep.* **7**, 40080; doi: 10.1038/srep40080 (2017).

**Publisher's note:** Springer Nature remains neutral with regard to jurisdictional claims in published maps and institutional affiliations.
